# *In Vitro* Glucuronidation of Ochratoxin A by Rat Liver Microsomes 

**DOI:** 10.3390/toxins5122671

**Published:** 2013-12-18

**Authors:** Zheng Han, Emmanuel K. Tangni, José Diana Di Mavungu, Lynn Vanhaecke, Sarah De Saeger, Aibo Wu, Alfons Callebaut

**Affiliations:** 1 Institute for Agri-food Standards and Testing Technology, Shanghai Academy of Agricultural Sciences, 1000 Jinqi Road, Shanghai 201403, China; E-Mail: hanzheng_ok@163.com; 2 Veterinary and Agrochemical Research Centre (CODA-CERVA), Unit of Toxins and Natural Components, Leuvensesteenweg 17, Tervuren B-3080, Belgium; E-Mails: Emmanuel.Tangni@coda-cerva.be (E.K.T.); alfons.callebaut@coda-cerva.be (A.C.); 3 Laboratory of Food Analysis, Faculty of Pharmaceutical Sciences, Ghent University, Harelbekestraat 72, Ghent B-9000, Belgium; E-Mails: Jose.DianaDiMavungu@UGent.be (J.D.D.M.); Sarah.DeSaeger@UGent.be (S.D.S.); 4 Laboratory of Chemical Analysis, Faculty of Veterinary Medicine, Ghent University, Salisburylaan 133, Merelbeke B-9820, Belgium; E-Mail: Lynn.Vanhaecke@UGent.be

**Keywords:** ochratoxin A, glucuronidation, metabolic pathway, rat liver microsomes, Orbitrap

## Abstract

Ochratoxin A (OTA), one of the most toxic mycotoxins, can contaminate a wide range of food and feedstuff. To date, the data on its conjugates via glucuronidation request clarification and consolidation. In the present study, the combined approaches of ultra high performance liquid chromatography-tandem mass spectrometry (UHPLC-MS/MS), UHPLC-Orbitrap-high resolution mass spectrometry (HRMS) and liquid chromatography-multiple stage mass spectrometry (LC-MS^n^) were utilized to investigate the metabolic profile of OTA in rat liver microsomes. Three conjugated products of OTA corresponding to amino-, phenol- and acyl-glucuronides were identified, and the related structures were confirmed by hydrolysis with β-glucuronidase. Moreover, OTA methyl ester, OTα and OTα-glucuronide were also found in the reaction solution. Based on these results, an *in vitro* metabolic pathway of OTA has been proposed for the first time.

## 1. Introduction

Ochratoxin A (OTA), one of the most potent mycotoxins in cereals and related products, is a secondary metabolite produced by several *Aspergillus* species notably *A. melleus*, *A. auricomus*, *A. alliaceus*, *A. petrakii*, as well as *Penicillium verrucosum* [1]. Because of its acute toxicity like nephrotoxicity, hepatotoxicity, teratogenicity and immunosuppression [2,3,4,5,6,7], OTA has been speculated to be associated with chronic renal diseases in humans (Balkan endemic nephropathy, interstitial nephritis) [8,9,10,11] and was designated by the International Agency for Research on Cancer (IARC) as a possible human carcinogen (group 2B) [12]. Based on animal experiments and epidemiological studies on humans, OTA has been regulated by legal limits in many countries [13]. A tolerable daily intake (TDI) of 5 ng/kg b.w. was derived for OTA on the basis of the lowest observed adverse effect level [14].

Contamination of food and animal feed with OTA may result in the presence of residues in edible offal and blood serum [15]. Despite efforts to reduce the amount of this mycotoxin in food and animal feed, a certain degree of contamination seems unavoidable at present. Also, OTA has been found regularly in human biofluids showing the exposure of humans to this mycotoxin [16,17]. 

There is a need for a deeper and more comprehensive understanding of toxicological findings in animals and extrapolation of the resulting data as reference to human. In this regard, in recent years many *in vitro* and *in vivo* kinetics and metabolic studies have been performed to provide information on its toxicology and food safety assessment [18,19,20]. OTA can be transformed by hydrolysis, hydroxylation and oxidation into a series of metabolites, *i.e.*, OTα, 4-OH-OTA, opened-OTA and ochratoxin quinone [19,21,22,23,24,25,26]. Moreover, one of the most important metabolisation reactions is glucuronidation, mainly in liver microsomes. Some other mycotoxins have been thoroughly investigated. Deoxynivalenol (DON) can be metabolized into DON-3-glucuronide and DON-15-glucuronide, which account for about 91% of total DON excreted in human urine [27]. The excretion rate of zearalenone (ZEN) was about 9.4%, also mainly as glucuronide [28,29]. These glucuronides have been successfully employed as biomarkers for assessment of human mycotoxin exposure [30,31]. With regard to OTA, at the cellular level, endogenous glucuronic acid can be conjugated to the phenolic group or carboxylic group under the catalytic reaction by uridine-diphosphate glucuronosyltransferase (UGT), which is a membrane bound microsomal enzyme adjacent to CYP [20]. OTA-conjugates have been detected in liver (8%–17%) and intestinal tissue (6%) [32]. Interestingly, the presence of glucuronide conjugates was also reported in bile of pigs upon feeding with OTA contaminated feed [33]. However, in all these reports, OTA-glucuronides were determined indirectly by using β-glucuronidase hydrolysis. No direct or definite evidence for the formation of OTA-glucuronides as well as complete chemical configurations are available. For example, Gross-Steinmeyer *et al.* did not observe any OTA glucuronide conjugates in *in vitro* experiments with rat and human hepatocytes [34]. 

These *in vivo* and *in vitro* data indicate that relevant data on OTA glucuronidation are still missing. Therefore, the purpose of this present work was to further clarify the formation of OTA metabolites by rat microsomal preparations and to clearly identify the structures of these metabolites to propose the major metabolic pathways of OTA in rat liver microsomes.

## 2. Results and Discussion

**Figure 1:**
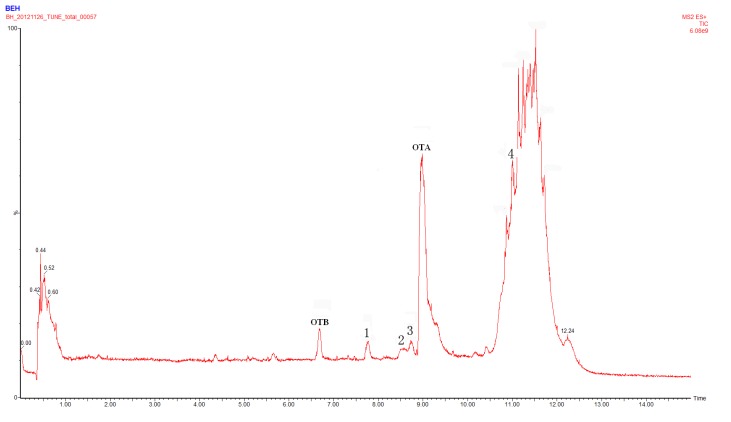
Full scan chromatograph of the Reaction 1 solution in positive electrospray ionization (ESI^+^) mode.

Metabolic profiling, focusing on metabolites of targeted analytes, provides important information on *in vivo* biotransformation, which is useful in toxicity testing. To identify the metabolites of OTA, liquid chromatography–mass spectrometry LC-MS analyses of reaction mixtures 1, 2, 3 and 4 were performed first. The full scan chromatogram of the Reaction 1 solution under positive electrospray ionization mode (ESI^+^) mode is shown in Figure 1 and is similar to that of Reaction 2, which indicates that alamethicin does not affect the yield of OTA glucuronides in the current reaction even though it should be used in the glucuronidation of T-2 and HT2 [35]. Four new peaks with retention times at 7.751 min (Compound 1), 8.536 min (Compound 2), 8.733 min (Compound 3) and 11.012 min (Compound 4) were evident while they could not be found in control incubations (Reactions 3 and 4). Special attention should be paid to the fact that OTB existed in Reactions 1, 2 and 3 with the same peak area. After direct injection of the standard solution of OTA, a similar amount of OTB was also detected, suggesting that OTB was the impurity in the standard solution but not formed by dechlorination of OTA.

### 2.1. Compound *1*

The negative full-scan mass spectrum showed a signal at *m/z* 578, which corresponds to the deprotonated molecule. Exact mass measurement of this signal (Table 1) provided the ion formula C_26_H_25_O_12_NCl.

**Table 1:** Ultra performance liquid chromatography-high resolution mass spectrometry (UHPLC-HRMS) data obtained from the four metabolites in HESI^+^ and HESI^−^ mode. Compounds, polarity, chemical configuration, observed masses, calculated masses and mass error of the molecular are reported.

Compound	Polarity	Ion formula	Observed m/z (Da)	Calculated m/z (Da)	Mass error (ppm)
1-3	+	C_26_ H_27_ O_12_ N Cl	580.12042	580.12219	−0.59
1-3	−	C_26_ H_25_ O_12_ N Cl	578.10812	578.10651	2.14
4	+	C_21_ H_21_ O_6_ N Cl	418.10428	418.10574	−0.91
4	−	C_21_ H_19_ O_6_ N Cl	416.09120	416.09009	1.66

**Figure 2:**
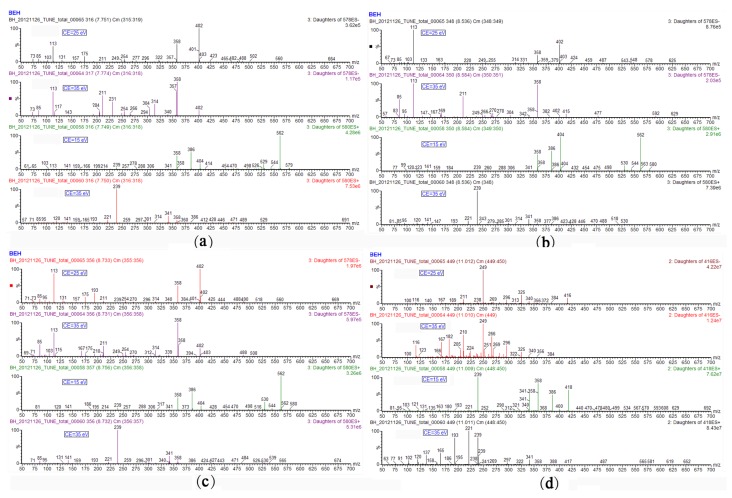
Product ion spectra of Compound 1 (**a**); Compound 2 (**b**); Compound 3 (**c**); and Compound 4 (**d**) using ultra performance liquid chromatography-tandem mass spectrometry (UPLC-MS/MS).

Structural elucidation of this compound was accomplished through product ion measurements by UHPLC-MS/MS analysis, and MS^n^ experiments by LC ion trap MS. The product ion spectrum of [M − H]^−^ (Figure 2a) showed four fragments at *m/z* 402, 358, 175 and 113. The fragments at *m/z* 175 and 113 are known to be diagnostic ions of glucuronate moieties [36], so it is reasonable to suppose that Compound 1 should correspond to a glucuronidated OTA. The fragment with *m/z* 402 is the negative ion [M − H]^−^ of OTA. The fragment ion at *m/z* 358 results from a neutral loss of CO_2_, revealing the presence of a carboxylic group, which is in agreement with previous data for OTA fragmentation [21,37,38,39].

Positive full-scan spectra of Compound 1 showed the ion at *m/z* 580 corresponded to [M + H]^+^, which was also confirmed by UHPLC-HRMS measurements, as shown in Table 1.

**Figure 3:**
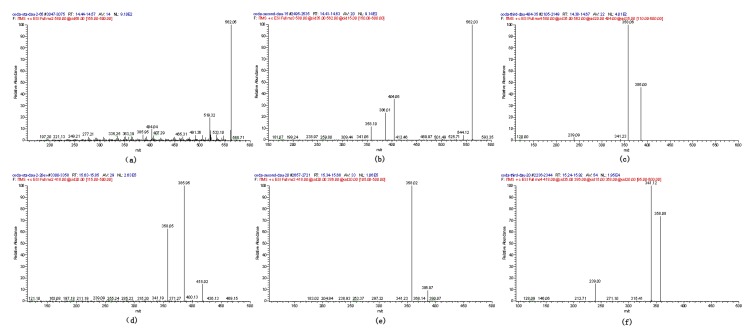
MS^n^ spectra of Compounds 1–3 (a, b, c) and Compound 4 (d, e, f). (**a**) MS^2^ (+) 580; (**b**) MS^3^ (+) 580→562; (**c**) MS^4^ (+) 580→562→404; (**d**) MS^2^ (+) 418; (**e**) MS^3^ (+) 418→386; (**f**) MS^4^ (+) 418→386→358 using LC ion trap MS.

The product ion spectrum of [M + H]^+^ showed different fragments. The protonated glucuronide first lost water and formed the fragment with *m/z* 562. Commonly, the product ion scan of glucuronide conjugates by LC-MS/MS provides the neutral loss of 176 Da (dehydrated glucuronic acid) due to the cleavage of glycosidic bond, with charge retention by the aglycone moiety. Therefore, as shown in Figure 3a, the mass difference between the ions 580 and 404 (protonated OTA ion), as well as between the fragments 562 and 386, corresponded to 176. The protonated ion at *m/z* 386, through losing CO, formed the fragments at *m/z* 358 (Figure 3a). 

### 2.2. Compounds *2* and *3*

The negative full scan showing the deprotonated molecules (*m/z* 578) of Compounds 2 and 3 were isobaric with the [M − H]^−^ of Compound 1, and therefore exhibited the same ion formula C_26_H_25_O_12_NCl (Table 1). The product ion spectrum of [M − H]^−^ also showed the same fragments of *m/z* 402, 358, 175 and 113. Meanwhile, under the ESI^+^ mode, the same data on the full scan and product ions indicated that Compounds 1, 2 and 3 were isomers, which all belonged to the OTA glucuronide conjugates. 

### 2.3. Acylic, Phenolic and Amino Glucuronides

OTA has three possible glucuronidation sites; in fact, it could form a phenol-, an acyl- or an amino-glucuronide. As shown in Figure 1, only small amounts of Compounds 1–3 were found indicating the low transformation rate from OTA to OTA-glucuronides. All three compounds were unknown and in order to characterize these isomers, different paths including retention times, literature references, MS/MS spectra and non-specific hydrolysis with β-glucuronidase were exploited in this work.

In the chromatographic separation (Figure 1), Compound 1 eluted at a retention time of 7.751 min, 0.785 min less than Compound 2 and 0.978 min less than Compound 3. This means that Compound 1 is the most polar, Compound 2 a bit less and Compound 3 the least polar. From the structure, OTA amino-glucuronide contained one carboxylic group and one phenolic hydroxyl group, so it should be more polar than OTA phenol-glucuronide or acyl-glucuronide. Compared to OTA phenol-glucuronide containing one carboxylic group, OTA acyl-glucuronide should be less polar due to only one phenolic hydroxyl group. Therefore, Compound 1 was proposed to be OTA amino-glucuronide, Compound 2 was OTA phenol-glucuronide and the last one was OTA acyl-glucuronide.

**Figure 4:**
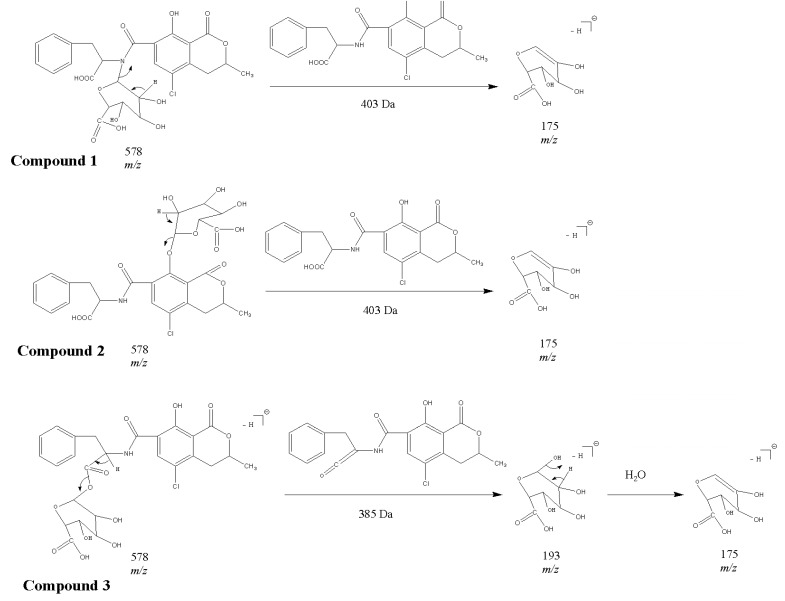
The proposed fragmentation pathways for Compounds 1–3.

The subsequent analyses of negative electrospray ionization mode (ESI^−^) product ions of the three compounds showed that besides the characteristic fragments (*m/z* 175 and 113) of glucuronate moieties, an intense peak at *m/z* 193 corresponding to the glucuronate anion was observed in the spectra of Compound 3 (Figure 2c). The proposed fragmentation pathways of the three glucuronide conjugates are shown in Figure 4. For Compound 1 and Compound 2, the ions at *m/z* 193 could not be found; as a consequence, the proposed pathway also assigned the structure of acyl-glucuronide to Compound 3. Similar results have been obtained in the previous reports for the other glucuronide conjugates, according to which, in the negative MS^2^ spectrum of acyl-glucuronide conjugates, the base peak fragment was the glucuronate anion at *m/z* 193 [36,40,41,42]. To the best of our knowledge, this is the first report to provide direct evidence for OTA-glucuronide generation.

Hydrolysis of the glucuronide conjugates was performed to provide more information for definite identification of the three compounds. The enzyme could hydrolyze the amino-, phenol- and acyl-glucuronide. The contents of the targeted analytes were accurately quantified by the established LC-MS/MS using the standard purified version in the present study. The parameters and collision energies of precursor ions and product ions selected for the analysis are listed in Table 2. The results demonstrated that after hydrolysis by β-glucuronidase, a significant signal decrease was clearly observed for all three compounds with similar extents, indicating their glucuronide forms (Figure S-1, Supplementary Data).

**Table 2:** The parameters and collision energies of precursor ions, product ions for the targeted analytes.

Names	Precursor ion (m/z)	Primary product ion (m/z)	Collision energy (eV)	Secondary product ion (m/z)	Collision energy (eV)	Ionization mode
Compound 1–3	580	358	20	239	38	ESI^+^
OTA methyl ester	418	358	18	239	32	ESI^+^
OTα	257	221	20	102	40	ESI^+^

### 2.4. Identification of Compound 4

The positive and negative full-scan mass spectra analyzed showed the signal at *m/z* 580 and 578 that corresponded to the protonated and deprotonated molecules, respectively. Exact mass measurement of [M + H]^−^ and [M − H]^+^ (Table 1) provided the ion formula C_21_H_21_O_6_NCl and C_21_H_19_O_6_NCl. After analysis by LC ion trap MS, typical fragments at *m/z* 386, 358, 341 and 239 indicated that this compound was OTA methyl ester [37] (Figure 3d–f). 

Unambiguous confirmation of this compound was obtained by means of OTA methyl ester preparation and from mass spectral comparison. The multiple reaction monitoring (MRM) method was established using the obtained standards (Table 2). The transformation rate from OTA to OTA methyl ester was almost 95% (Figure S-2, Supplementary Data). Then, Reaction 1 and Reaction 2 solutions were analyzed by the established method, and OTA methyl ester was clearly identified. Although a little amount of OTA methyl ester could be found in Reaction 3, the quantity was less than 5% of that in Reaction 1 or 2, in addition to the fact that no OTA methyl was found in Reaction 4, indicating that OTA methyl was not an artifact but produced by this reaction (Figure S-3, Supplementary Data). The reaction conditions in the present study were favorable for the glucuronidation biotransformation resulting in the production of OTA-glucuronides, while esterification reaction in principle could not be catalyzed by microsomes. However, in the present study, it was clearly demonstrated that OTA methyl ester could be produced with relative high content. 

### 2.5. Identification of OTα

As reported, OTα is a very important metabolite [43,44]. However, it was not found in the reaction solution in full scan MS mode. A MRM method for detection of OTα was established by direct injection of the standard solution (1 μg mL^−1^), with the parameters indicated in Table 2. The solutions of Reactions 1 and 2 before and after hydrolysis were analyzed by the established MRM method. The results showed that a very low content of OTα was present in the solutions of Reactions 1, 2 and 3, however, after hydrolysis, the concentration of OTα in Reactions 1 and 2 significantly increased (Figure S-4, Supplementary Data), indicating the existence of OTα-glucuronide formed by the glucuronidation transformation. It is not surprising that OTα, a major metabolite *in vivo*, was hardly found in incubations with liver microsomes, since hydrolysis is known to occur in the gut. When peptide cleavage occurs by an unspecific hydrolytic activity in the reaction mixture, OTα is then apparently further conjugated by microsomal UGTs in Reactions 1 and 2. As hydrolysis but not conjugation could occur in Reaction 3, however, a low content of OTα was also found in Mixture 3.

### 2.6. Proposed Metabolic Pathways

**Figure 5:**
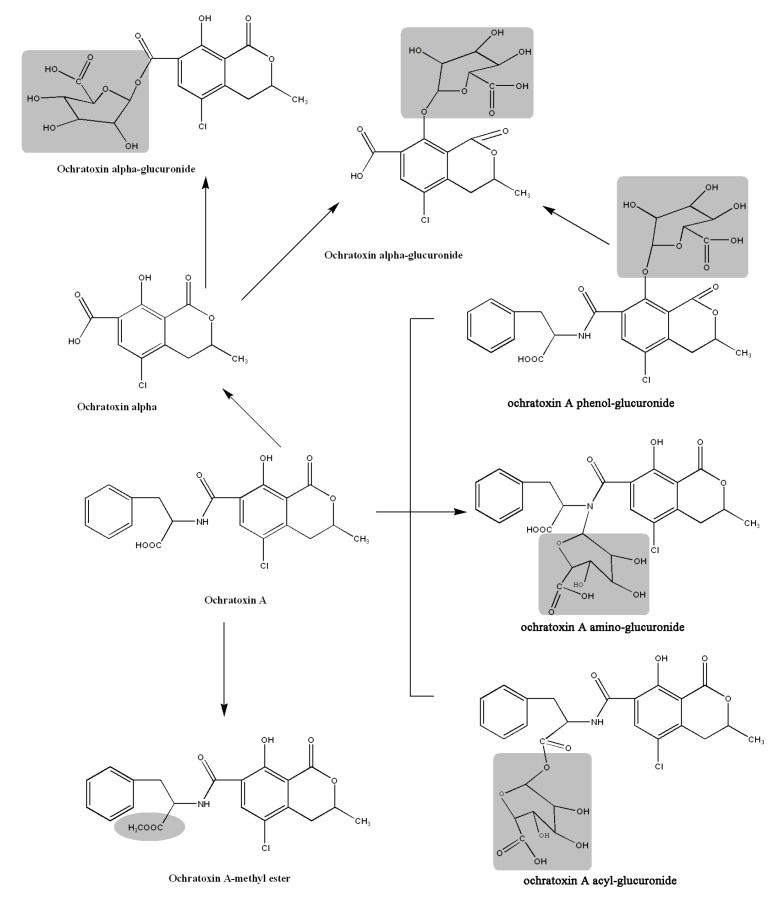
The proposed metabolic pathway for ochratoxin A (OTA) via glucuronidation by rat liver microsomes.

Based on the above observations, the *in vitro* metabolic profile of OTA via biotransformation by rat liver microsomes is proposed in Figure 5. First, the glucuronidation reaction could occur. There are three possible glucuronidation sites, and OTA has been transformed to phenol-, acyl- and an amino-glucuronide. Second, the Phase I methylation reaction could happen on OTA under such conditions, and a relatively high amount of OTA methyl ester was formed. Last but not least, hydrolysis could occur and OTA was conversed to OTα. The site of hydrolysis was tentatively proposed to be N-C9. Then, OTα was glucuronidated to form OTα-glucuronide. On the other side, OTA-glucuronides also might be directly hydrolyzed to form OTα-glucuronide. 

## 3. Experimental Section

### 3.1. Chemicals and Reagents

Methanol and acetonitrile were ultra-high performance liquid chromatography-mass spectrometry (UHPLC-MS) grade from Biosolve (Valkenswaard, the Netherlands). Water was purified by a Milli-Q system (Millipore, Brussels, Belgium). Ammonium acetate (AmAc), ammonium formate (AF), formic acid (FA), uridine 5’-diphosphoglucuronic acid trisodium salt (UDPGA), uridine 5’-diphospho-*N*-acetylgalactosamine disodium salt (UDPAG), anhydrous magnesium chloride (MgCl_2_) and 2-amino-2-(hydroxymethyl)-1,3-propanediol (Tris) were from Sigma-Aldrich (Saint Louis, MO, USA). β-glucuronidase and microsomes (pooled from male rat liver) were from Sigma-Aldrich (Saint Louis, MO, USA).

Ochratoxin A (OTA) and ochratoxin α (OTα) were from Coring System Diagnostix GmbH (Gernshein, Germany). Accurately weighed solid portions of OTA and OTα standards were dissolved in acetonitrile to prepare 0.5 mg mL^−1^ of stock solutions.

The OTA methyl ester was prepared according to the previous report with a minor modification [45]. The OTA standard solution (0.5 mL) was mixed with 9 mL of methanol and 0.5 mL of 12 N HCl, and incubated for 24 h at room temperature. Afterwards, 2.5 mL of extracts were dried under a nitrogen stream, redissolved with 1.5 mL of methanol and ready for analysis.

### 3.2. UHPLC-MS/MS Analysis

The LC system consisted of an Acquity UPLC^®^ H-class (Waters, Milford, MA, USA). The compounds were separated on an Acquity^®^ UPLC HSS T3 column (100 mm × 2.1 mm, 1.7 μm) at 40 °C, with a mobile phase flow rate of 0.5 mL min^−1^. The mobile phase consisted of (A) 10 mmol L^−1^ ammonium acetate solution, (B) water and (C) methanol. A linear gradient elution program was applied as follows: 0 min 1% A and 29% C, 10 min 1% A and 59% C, 10.2 min 1% A and 99% C, 11 min 1% A and 99% C, 11.8 min 1% A and 29% C, and hold on for a further 2.2 min for re-equilibration, giving a total run time of 13 min. The injection volume was 5.0 μL (partial loop with needle overfill). 

A XEVO TQ-S^®^ mass spectrometer (Waters) was used for the analysis of the target compounds. Full scan analysis was performed both in the positive electrospray ionization mode (ESI^+^) and the negative electrospray ionization (ESI^−^) mode. The following settings were used: source temperature, 150 °C; desolvation temperature, 500 °C, scan range, *m/z* 100–1000, inter-scan delay, 0.01 s. The cone and desolvation gas flows were 30 and 1,000 L h^−1^, respectively. Data acquisition and processing were performed using MassLynx v4.1 (Waters). 

### 3.3. UHPLC-Orbitrap-HRMS Analysis

UHPLC-Exactive^TM^ Benchtop Orbitrap mass spectrometer (Thermo Fisher Scientific, San José, CA, USA) analysis in the full scan mode (*m/z*, 100–1000) was utilized for the metabolic profiling study. Chromatographic separation was achieved on a Zorbax Eclipse Plus C_18_ column (100 mm × 2.1 mm, 1.8 μm) at 30 °C, with a mobile phase flow rate of 0.4 mL min^−^^1^. The mobile phase consisted of (A) water/methanol (5/95, v/v) containing 0.1% formic acid and 10 mM ammonium formate and (B) water/methanol (95/5, v/v) containing 0.1% formic acid and 10 mM ammonium formate. A linear gradient elution program was applied as follows: 0 min 0% B, 0.5 min 0% B, 20 min 99.1% B, 21 min 99.1% B, 24 min 0% B and hold on for a further 4 min for re-equilibration, giving a total run time of 28 min. The injection volume was 5.0 μL. The mass spectrometer was operated both in HESI^+^ and in HESI^−^. The following settings were used: spray voltage, 4.5 kV; capillary temperature, 250 °C; heater temperature, 250 °C; sheath gas flow, 45 a.u.; auxiliary gas, 10 a.u; sweep gas, 2 a.u., resolution 100000 FWHM at 1 Hz (1 scan per second). The automatic gain control (AGC) target was set at high dynamic range (3e^6^), and the maximum injection time was 20 ms. Initial instrument calibration was achieved by infusing calibration mixtures (Thermo Fisher Scientific) for positive and negative ion modes. The positive calibration mixture included caffeine, Met-Arg-Phe-Ala acetate salt (MRFA) and Ultramark 1621^®^, while the negative calibration solution comprised sodium dodecyl sulfate, sodium taurocholate and Ultramark 1621^®^. These compounds were dissolved in a mixture of acetonitrile, water and methanol, and both mixtures were infused with a Chemyx Fusion 100 syringe pump (Thermo Fisher Scientific). Data were acquired and processed by Xcalibur 2.1 and Sieve 2.0 software (Thermo Fisher Scientific, Brookfield, Los Angeles, CA, USA).

### 3.4. LC-Ion-Trap Analysis

HPLC-ion-trap-MS system (Thermo Fisher Scientific) was used for the fragments analysis of the targeted analytes. The column used was an X-bridge C18 column (3.5 μm, 2.1 × 150 mm), supplied by Waters (Milford, MA, USA). The mobile phase consisted of (A) water/methanol (5/95, v/v) containing 0.1% formic acid and 10 mM ammonium formate and (B) water/methanol (95/5, v/v) containing 0.1% formic acid and 10 mM ammonium formate. The linear gradient elution program for LC-Ion-trap analysis was: 0–1 min B = 50%, 1–13 min B = 50%–97%, 13–18 min B = 97%, 18–19 min B = 97%–50%, and hold on for a further 6 min for re-equilibration, giving a total run time of 25 min. The mass spectrometer was operated both in HESI^+^ and in HESI^−^ with the following settings: source voltage of 5 kV, capillary temperature of 250 °C; heater temperature of 175 °C; sheath gas flow of 45 a.u.; aux gas of 10 a.u. The Xcalibur 2.0.7 software (Thermo Scientific) was used for instrument control, data acquisition and processing.

### 3.5. Model Reactions

The *in vitro* metabolic study of OTA via glucuronidation biotransformation by rat liver microsomes (Sigma-Aldrich, Saint Louis, MO, USA) was performed based on a slightly modified procedure adopted from Wu *et al.* [46] and Welsch *et al.* [35] for synthesis of DON-glucuronides.

**Reaction 1**: An aliquot of OTA stock solution (241.8 μL, 0.3 μM) was transferred into a 5 mL tube and dried by nitrogen gas under 40 °C. Next, 15 μM of UDPGA, 0.25 μM of UDPAG and 5 μM of MgCl_2_ were added and all these reagents were dissolved in 400 μL of 50 mM Tris-HCl buffer (pH = 7.4). Rat liver microsomes (100 μL) were firstly mixed with 5 μg of alamethicin and kept on ice for 10 min before adding into the tubes. Then, the 500 μL reaction mixture was inverted a few times and incubated at 37 °C in a water bath for 1.5 h. The reaction was terminated by addition of 1 mL of methanol, vortexed for 30 s and centrifuged at 14,000 rpm for 10 min. The supernatant (100 µL) was diluted with 900 μL of methanol/water solution (20/80, v/v) and was ready for injection.

**Reaction 2**: An aliquot of OTA stock solution (241.8 μL, 0.3 μM) was transferred to a 5 mL tube and dried by nitrogen gas under 40 °C. Next, 15 μM of UDPGA, 0.25 μM of UDPAG and 5 μM of MgCl_2_ were added and all these reagents were dissolved in 400 μL of 50 mM Tris-HCl buffer (pH = 7.4). Then 100 μL (~2 mg of microsomal protein) of rat liver microsomes were added. The reaction mixture (500 μL) was subsequently pretreated as reaction 1.

**Reaction 3**: A blank reaction was performed using the same procedure and ingredients as in reaction 2, except for UDPGA. 

**Reaction 4**: A control reaction was also performed using the same procedure and ingredients as in Reaction 2, except for OTA. 

### 3.6. Hydrolysis of the Glucuronides

Hydrolysis of the reaction solution was performed for further identification of the OTA-glucuronides. An aliquot of the reaction solution (10 μL) was incubated with β-glucuronidase (1.5 units/reaction) (type IX, from *E. coli,* Sigma-Aldrich, Saint Louis, MO, USA) at 37 °C in 0.25 mL of 0.1 M NaAc buffer (pH = 5) for 18 h. The reaction was terminated by addition of 1 mL methanol, vortexed for 30 s and centrifuged at 14,000 rpm for 10 min. The supernatant was passed through a 0.22 μm nylon filter and was ready for injection 

## 4. Conclusions

To address the remaining uncertainties regarding OTA biotransformation by liver microsomes, UHPLC-MS/MS, UHPLC-Orbitrap-HRMS and LC-ion-trap were applied as combined strategies to investigate the metabolic profile of OTA via glucuronidation by rat liver microsomes. Three different OTA glucuronide conjugates, which corresponded to OTA amino-glucuronide, OTA phenol-glucuronide and OTA acyl-glucuronide were clearly identified. The suggested structures were supported by the fragments observed in the mass spectrometers and by hydrolysis with β-glucuronidase. OTA methyl ester, OTα and OTα-glucuronide were formed in the same reaction mixture. A possible *in vitro* biotransformation pathway of OTA in rat microsomes was proposed. The results obtained here will help to have deeper understanding on the theoretical basis of OTA clinical toxicology.

## Supplementary Files

Supplementary File 1 Supplementary Data (PDF, 525 KB)
