# Supplementary material for: In Vitro Glucuronidation of Ochratoxin A by Rat Liver Microsomes
Source: Toxins (Basel). 2013 Dec 18;5(12):2671–85. doi: 10.3390/toxins5122671 (PMC3873705; doi:10.3390/toxins5122671)
Supplement: Supplementary File 1 — Supplementary Data (PDF, 525 KB) [file toxins-05-02671-s001.pdf]

Supplementary Data

Figure S-1. Comparison of the contents of three OTA glucuronide conjugates in the Reaction 2 solution untreated (**a**,**b**) and the solution hydrolyzed with β-glucuronidase (**c**,**d**).

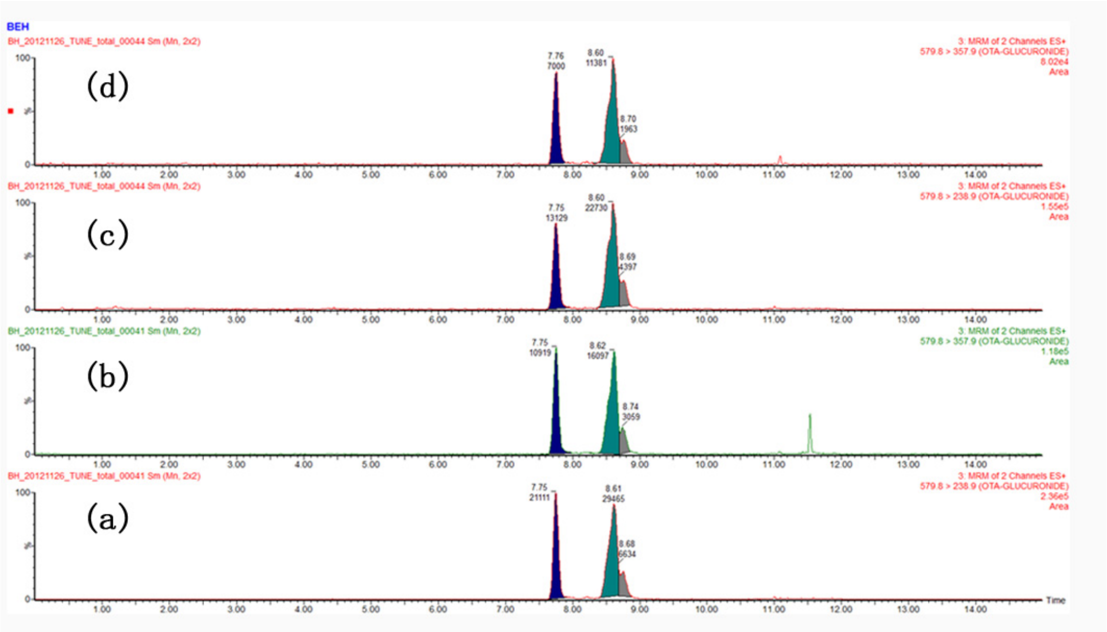

Figure S-2. Comparison of the contents of OTA and OTA methyl ester in the solutions before methylation (**A**,**B**) and after methylation (**a**,**b**).

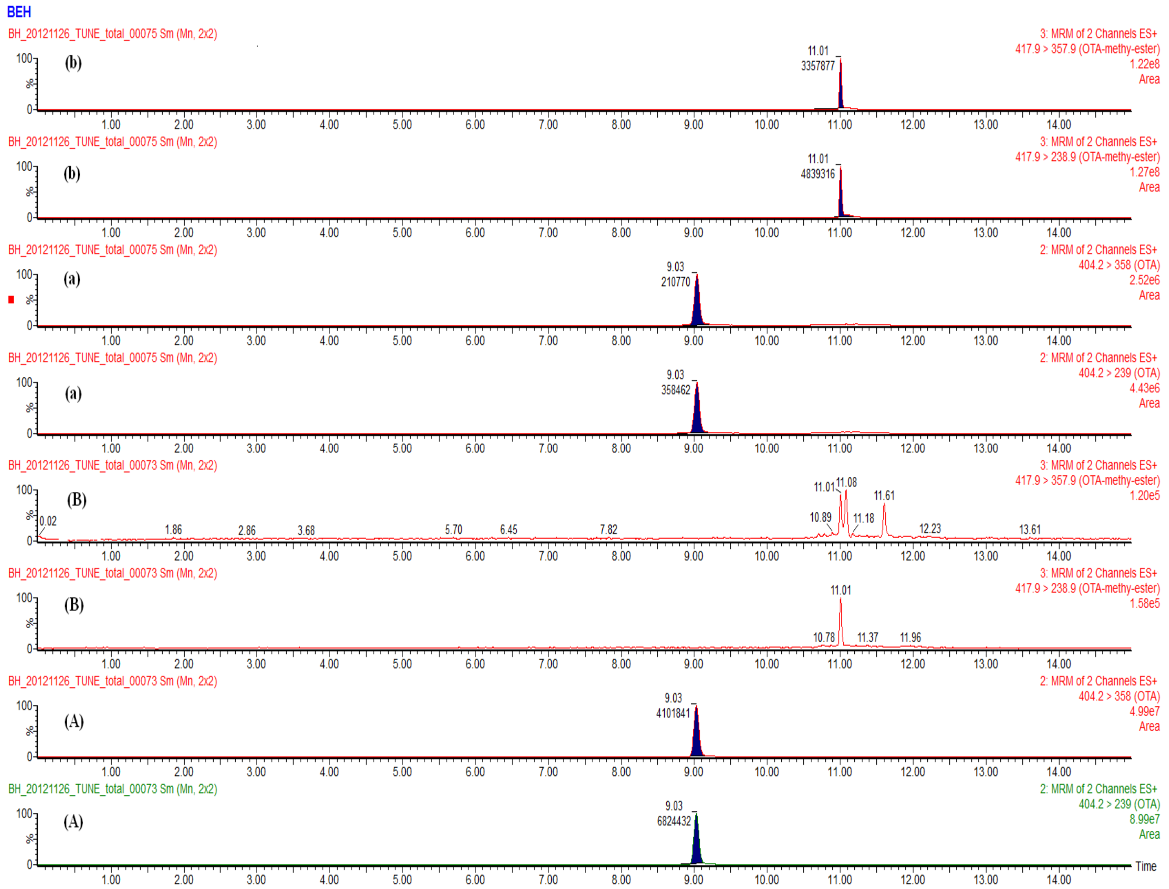

Figure S-3. Comparison of the contents of OTA methyl ester in different reaction solutions.

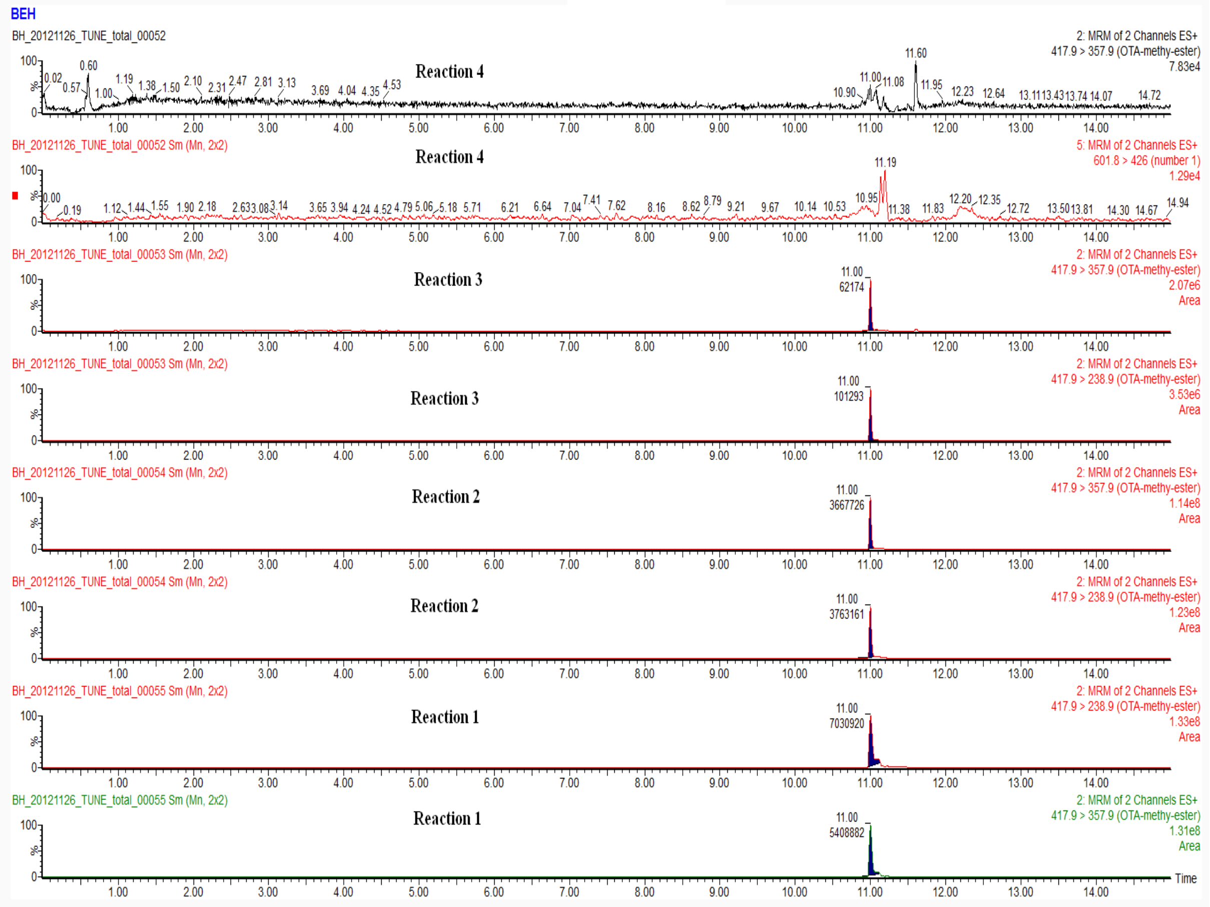

Figure S-4. Comparison of the contents of OTα in the Reaction 2 solution untreated (**a**) and in the solution hydrolyzed with β-glucuronidase (**b**)

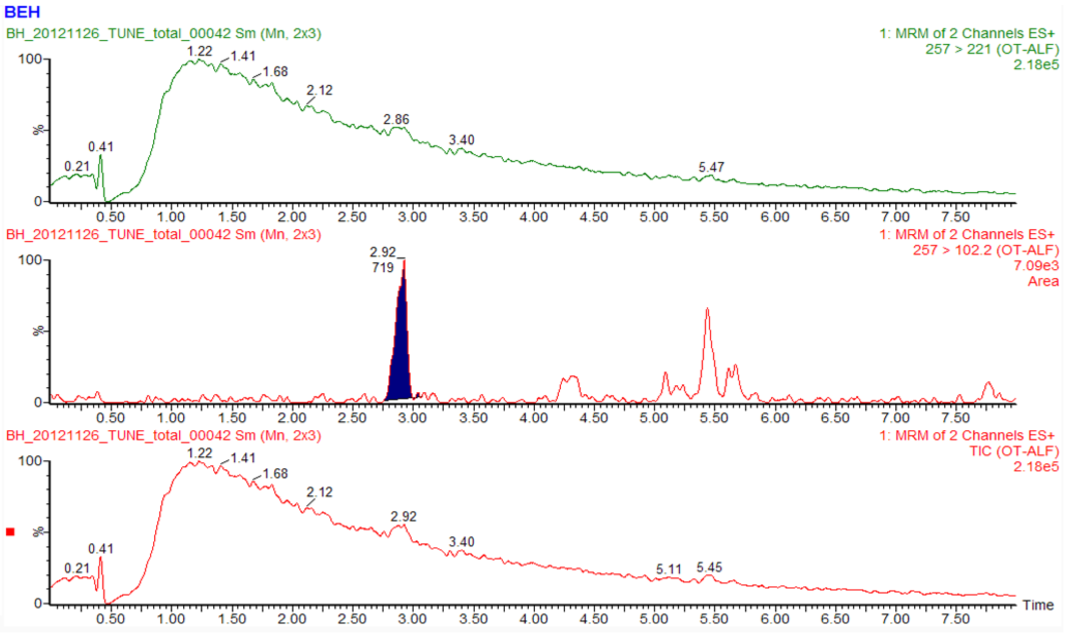

(**a**)

Figure 4-S. *Cont.*

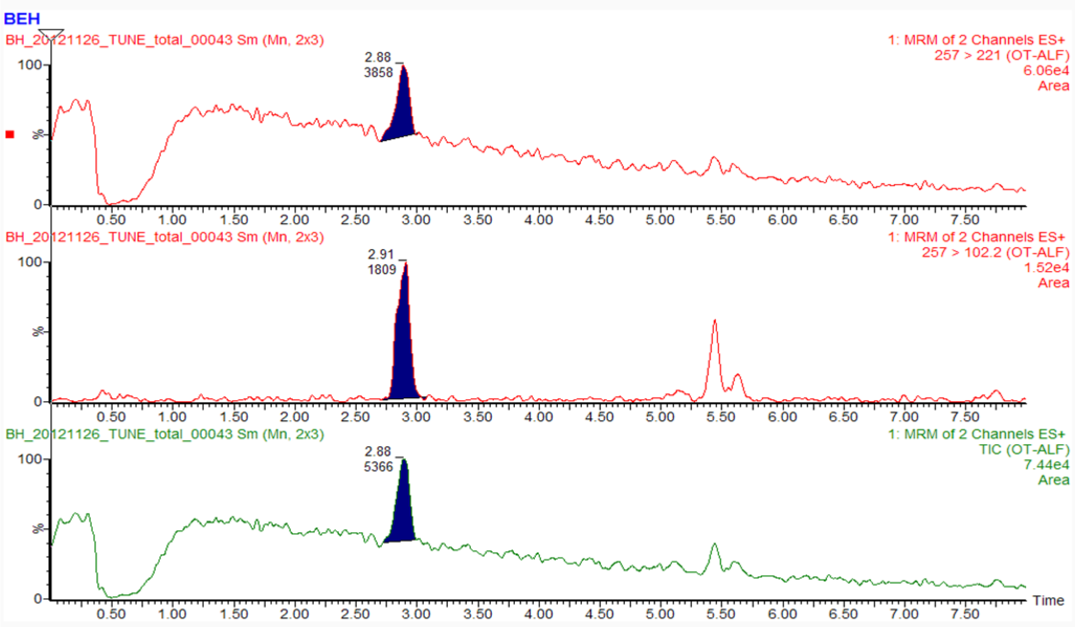

(**b**)
